# Estimating At-Sea Mortality of Marine Turtles from Stranding Frequencies and Drifter Experiments

**DOI:** 10.1371/journal.pone.0056776

**Published:** 2013-02-20

**Authors:** Volker Koch, Hoyt Peckham, Agnese Mancini, Tomoharu Eguchi

**Affiliations:** 1 Department of Marine Biology, Universidad Autónoma de Baja California Sur, La Paz, Baja California Sur, Mexico; 2 Investigación para la Conservación y el Desarrollo, La Paz, Baja California Sur, Mexico; 3 Department of the Directorate, Leibniz Center for Tropical Marine Ecology, Bremen, Germany; 4 Grupo Tortuguero de Las Californias AC, La Paz, Baja California Sur, México; 5 Center for Ocean Solutions, Stanford University, Pacific Grove, California, United States of America; 6 Boomerang For Earth Conservation, Antony, France; 7 Protected Resources Division, Southwest Fisheries Science Center, National Marine Fishery Service, NOAA, La Jolla, California, United States of America; University of Wales Swansea, United Kingdom

## Abstract

Strandings of marine megafauna can provide valuable information on cause of death at sea. However, as stranding probabilities are usually very low and highly variable in space and time, interpreting the results can be challenging. We evaluated the magnitude and distribution of at-sea mortality of marine turtles along the Pacific coast of Baja California Sur, México during 2010–11, using a combination of counting stranded animals and drifter experiments. A total of 594 carcasses were found during the study period, with loggerhead (62%) and green turtles (31%) being the most common species. 87% of the strandings occurred in the southern Gulf of Ulloa, a known hotspot of loggerhead distribution in the Eastern Pacific. While only 1.8% of the deaths could be definitively attributed to bycatch (net marks, hooks), seasonal variation in stranding frequencies closely corresponded to the main fishing seasons. Estimated stranding probabilities from drifter experiments varied among sites and trials (0.05–0.8), implying that only a fraction of dead sea turtles can be observed at beaches. Total mortality estimates for 15-day periods around the floater trials were highest for PSL, a beach in the southern Gulf of Ulloa, ranging between 11 sea turtles in October 2011 to 107 in August 2010. Loggerhead turtles were the most numerous, followed by green and olive ridley turtles. Our study showed that drifter trials combined with beach monitoring can provide estimates for death at sea to measure the impact of small-scale fisheries that are notoriously difficult to monitor for by-catch. We also provided recommendations to improve the precision of the mortality estimates for future studies and highlight the importance of estimating impacts of small–scale fisheries on marine megafauna.

## Introduction

Carcasses of marine turtles and marine mammals encountered on shorelines (strandings) can provide valuable information on minimum mortality at sea and also about cause of death, if the animals arrive fresh and can be necropsied [1]. However, probability of stranding varies widely in space and time, and usually does not exceed 10–20% of total mortality even in near-shore waters, as predators, scavengers, wind and currents prevent carcasses from reaching the shore [2], [3], [4]. At greater distances from shore stranding probability diminishes even more and animals that die offshore may never strand. It is therefore extremely difficult to estimate total mortality when using stranding frequencies only, even in near-shore waters [2], [3], [4].

However, knowledge of mortality from natural events or anthropogenic threats is important for the conservation and management of marine organisms [5], [6], [7], [8]. All sea turtle species, and many marine mammals and sea birds are listed on the IUCN Red List [9], and many suffer high mortalities from fisheries bycatch or direct harvest [4], [6], [10], [11], [12], [13], [14].

Baja California Sur (BCS) is one of the areas in the world with the highest reported stranding frequencies of marine turtles and mammals [4], [10], [12], [15]. Bycatch in coastal gillnet and long-line fisheries [11], [16] and poaching [10], [16], [17], [18], [19] have been identified as a major causes of sea turtle deaths in the area. Small-scale fisheries have recently been identified as a major source of sea turtle bycatch in several countries, possibly causing even higher impacts than industrial fisheries [4], [11], [12], [16], [20], [21], [22], [23]. Bycatch rates are greater when there is an overlap between fishing areas and important sea turtle habitat [4], [11], [14]. Bycatch mortality may be a driving force for population declines in sea turtles and other marine megafauna [6] and small-scale fisheries that are often little studied have much more impact globally than previously thought [10], [11], [14], [18], [11], [23,24].

Several estimates for sea turtle mortality caused by small-scale fisheries have been reported for some areas in BCS: Nichols [25] estimated 10,000–30,000 sea turtle deaths per year for the whole region through examinations of strandings and interviews of local people; Mancini et al. [16], [19] determined sea turtle consumption through interviews in fishing communities; Peckham et al. [12] extrapolated bycatch levels from certain fisheries; and Mancini et al. [4] compared mortality estimates from interviews and from modelling stranding probabilities of drifters with the actual stranding frequencies of carcasses.

Coastal fisheries vary considerably on small spatial and temporal scales, both in intensity and gear [26]. Sea turtle distribution is also highly variable, both in space and time, and fishermen and sea turtles often occur in the same areas at the same time, to the detriment of the latter. Fisheries management must be adapted locally to protect endangered species in areas where significant overlap with fisheries occur, and where certain gear types may cause high bycatch mortality [4], [8], [11], [21], [22], [27], [28], [29], [30], [31]. It is important to identify these hotspots [32] to arrive at estimates of total mortality, which can be used for risk assessment and population viability models that determine the impact of the mortality on the species. Here, we (1) evaluated the relative magnitude and distribution of loggerhead strandings on the Pacific coast of BCS 2010–11, (2) estimated the stranding probability of sea turtles that die as bycatch at four fishing areas for small-scale fisheries in BCS by using drifters during the peak fishing season, (3) estimated the total sea turtle mortality from the observed stranded carcasses and estimated stranding probabilities at four index sites along the Pacific coastline of BCS.

### Study Area

**Figure 1:**
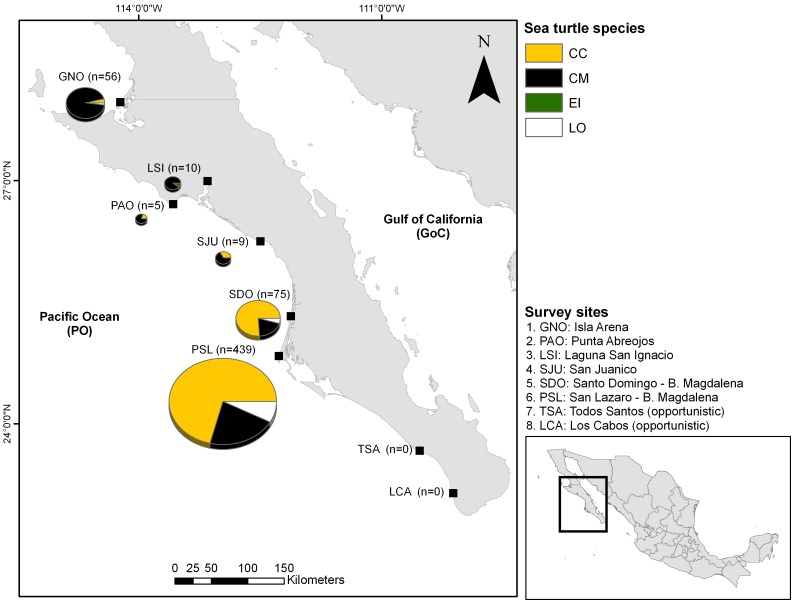
Cumulative sea turtle strandings at the Pacific coast of BCS during 2010 and 2011. Round markers are sites that were monitored specifically for carcasses; rectangles are nesting beaches of olive ridley turtles that were opportunistically monitored for carcasses. Shoreline length (measured as coastline length along the high tide mark) and site names are shown.

The Pacific coast of Baja California Sur (BCS) consists of more than 1000 km of coastline between 23° and 28°N, with several interspersed coastal lagoon systems that are important as artisanal fishing grounds and as nursery and feeding areas for many species [10], [25]. The region is located at the southern end of the highly productive California Current and characterized by year-round coastal upwelling conditions with mesoscale eddies, and fronts with seasonally variable sea surface temperature (SST) (15 to 26°C), and high chlorophyll *a (chl a)* concentrations (0.2 to 19.0 mg m^–3^) [32], [33], [34], [35]. Sea surface temperature varies between 14–23°C in the north and 18–30°C in the south. Salinity is 35 PSU or higher, due to the arid climate. During most of the year, northwesterly winds prevail. The sampling sites in this study are shown in Figure 1. The large bay formed between PSL in the south and PAO in the north is called Gulf of Ulloa, and is a known hotspot for loggerhead sea turtles [11].

The state of Baja California Sur, Mexico, has about 500,000 inhabitants with 3,633 artisanal fishing boats registered, of which more than half operate along the Pacific coast. Artisanal fisheries mostly target finfish (approximately 74,146 tons of disembarked weight), but also shellfish (approx. 18,203 tons), sharks (approx. 7,382 tons) and shrimp (approx. 1,205 tons) [36]. Most boats operate with gillnets, less with long-lines and traps, and the shrimp fishery operates with small trawl nets, while most shellfish is taken by divers (using hooka) [36], [37].

## Methods

### Beach Monitoring

We monitored beaches for stranded carcasses with an all-terrain vehicle, except in Playa San Lazaro (PSL), where a 4-wheel-drive vehicle was used. Sampling was conducted monthly in Guerrero Negro at Isla Arena (GNO), Punta Abreojos (PAO), San Juanico (SJU) and Santo Domingo (SDO), and daily (May-September) and biweekly (October-April) in PSL. At the two southern sites Todos Santos (TSA) and Los Cabos (CSL), collaborators monitored nesting beaches daily during the nesting season of olive ridley turtles (July-November), and approximately monthly during the rest of the year. We recorded every stranded turtle encountered, measured curved carapace length (CCL), photographed and determined the cause of death when possible [10]. Then we marked the carcass with spray paint and cable binders or a piece of rope to avoid recounting it in the future. At PSL carcasses were removed from the beach. For further methodological details of carcass sampling, please refer to Mancini et al. [4], Koch et al. [10] and Peckham et al. [12]. The necessary permits for this study were granted by “Dirección General de Vida Silvestre/Secretaría para el Medio Ambiente y los Recursos Naturales (SEMARNAT)” in Mexico, permit numbers: SGPA/DGVS/05603/09, SGPA/DGVS/08187/10.

### Estimation of Stranding Probabilities

Drifter deployments were conducted during the summer and fall of 2010 using a ∼7 m skiff in the main fishing areas off Bahía Magdalena (PSL, SDO), San Juanico (SJU), Punta Abreojos (PAO), and Isla Arena (GNO). Deployments were always done with local fishermen who knew where the fleet was working at the time to ensure coverage of the fishing area. The same process was repeated in summer and fall of 2011, but only in the area of Bahía Magdalena where >90% of the strandings along the coast took place. Individually marked drifters (oranges, branded with a unique code on the skin using a soldering iron) were deployed along predetermined transect lines at regular intervals of 0.3–1 km and their individual release locations recorded. Beaches were monitored every day for 4–7 days during and after drifter deployments to recover stranded carcasses and drifters. Monitoring was stopped when no drifters had been found for two consecutive days. Two drifter experiments were conducted at each area (except at GNO, n = 1) with different tidal and wind conditions, to encompass representative conditions during summer and fall.

To estimate probability of stranding at each study site, we developed a hierarchical statistical model for the stranding process. We assumed that drifters would behave similarly to turtle carcasses while drifting at the ocean surface [4]. Oranges have a slightly lower density than seawater [38], and less than 5–10% of their volume is above the water, so they would follow very closely the wind-driven surface current in the uppermost layer. This is similar to buoyant sea turtle carcasses, thus should mimic their drift behavior closely. This is very different to normal Lagrangian drifters which have a sea anchor at 15 m depth [39], and are thus not representative for surface currents in the first 10–20 cm. Deployment locations were grouped into quadrats (*j*  = 1, …, *J*) and the size of each quadrat was determined through trial and error to obtain a sufficient number of deployments from each quadrat. The size ranged from 0.01°×0.01° to 0.05°×0.05°, approximately 1.1×1.1 km to 5.6×5.6 km respectively. The number of drifters that stranded ashore from the *j* ^th^ quadrat (*m_j_*) was modeled with a binomial distribution with the total number of deployed oranges from the quadrat (*N_j_*) and the quadrat-specific stranding probability (*p_j_*).
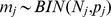



The quadrat-specific stranding probabilities were assumed to come from a hyper-distribution, which was modeled with a Beta distribution:
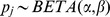
where the site-specific overall stranding probability (*p*) was inferred from this beta distribution. We used the mode of the beta distribution as the point estimate whereas the 95% posterior interval was used to express uncertainty in the estimate. Probability of detection of drifters on the beaches was assumed to be constant over all study locations because the same personnel and same methods were used to search for stranded oranges at each site.

### Estimating Total Mortality at Sea

Site-specific stranding probabilities (*p*) and the observed number of carcasses at beaches were used to estimate the total deaths at sea. We used two different approached to model the number of observed carcasses. For the first approach, the observed number of carcasses (*c*) within the same stretch of a coast line where drifters washed ashore was modeled as a binomial distribution with the unknown total number of carcasses (*C_total_*), including those that were not stranded, and the site-specific stranding probability (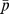), where 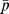 was the mode of the hyper-distribution of *p*’s:
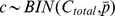



Posterior distributions of *α’s and β*’s of the beta distribution from the aforementioned drifter model were used to obtain uncertainty in 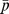, which was expressed in a 95% probability interval, whereas the mean was used as a point estimate. Following Raftery [40], we assumed *C_total_* has a Poisson distribution with mean μ:
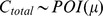



We used a gamma prior distribution on μ with variance  = 100 and mean = the number of observed carcasses *(c).* To determine the effects of the prior gamma distribution, the analysis was repeated with a different gamma prior distribution with variance 50 and the same mean and results compared.

For the second approach (following a suggestion by an anonymous reviewer) rather than treating the total number of carcasses as an unknown parameter, we modeled the number of un-stranded carcasses (*u*) as a negative binomial random variable:
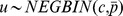
where *u* is the number of carcasses that were not stranded. For this approach, *C_total_* = *c*+*u*.

To apply drifter stranding probabilities to turtle carcasses, we used the carcass data that spanned a 15-day period centered at the middle of drifter deployment dates. For example, if drifters were deployed between 15^th^ and 18^th^ of July 2010 at PSL, the total deaths were estimated for the period between 9^th^ and 23^rd^ of July 2010.

We used a Bayesian approach with vague prior distributions on all parameters to estimate stranding probabilities. To obtain a joint posterior distribution of the parameters, OpenBugs [41] was run through matbugs (available from http://code.google.com/p/matbugs/) in Matlab (v. R2011b; MathWorks). Five independent chains of at least 20,000 steps were used to tune the Markov chain Monte Carlo sampling parameters, followed by 100,000 to 500,000 steps of sampling. These chains were thinned every five steps to reduce auto-correlations. Summary statistics were computed from the remaining samples.

## Results

In total, over 1500 hours of sampling effort were used to patrol more than 13,000 km of shoreline during 2010 and 2011. A total of 594 turtle carcasses were recovered during the surveys, of which 370 (62% of the total) were loggerhead turtles, 186 (31%) were green turtles, 34 (6%) were olive ridley turtles, and 1 (<0.2% of the total) was a hawksbill turtle (Figure 1). Species compositions shifted from loggerhead turtles in the south to green turtles in the northern part of the study area. The number of stranded turtles was highest at PSL where 74% (439 turtles) of the total strandings were recorded, followed by SDO with almost 13% (75 turtles) and GNO with 9% (56 turtles). No stranded turtles were reported from the two nesting sites of olive ridley and leatherback turtles, Todos Santos and Cabo San Lucas. Only 10 carcasses (1.8%) showed clear signs of fishing gear (hooks/net marks, entanglement). Cause of death could not be determined in the other carcasses due to lack of marks, presence of scavengers and advanced decomposition.

**Figure 2:**
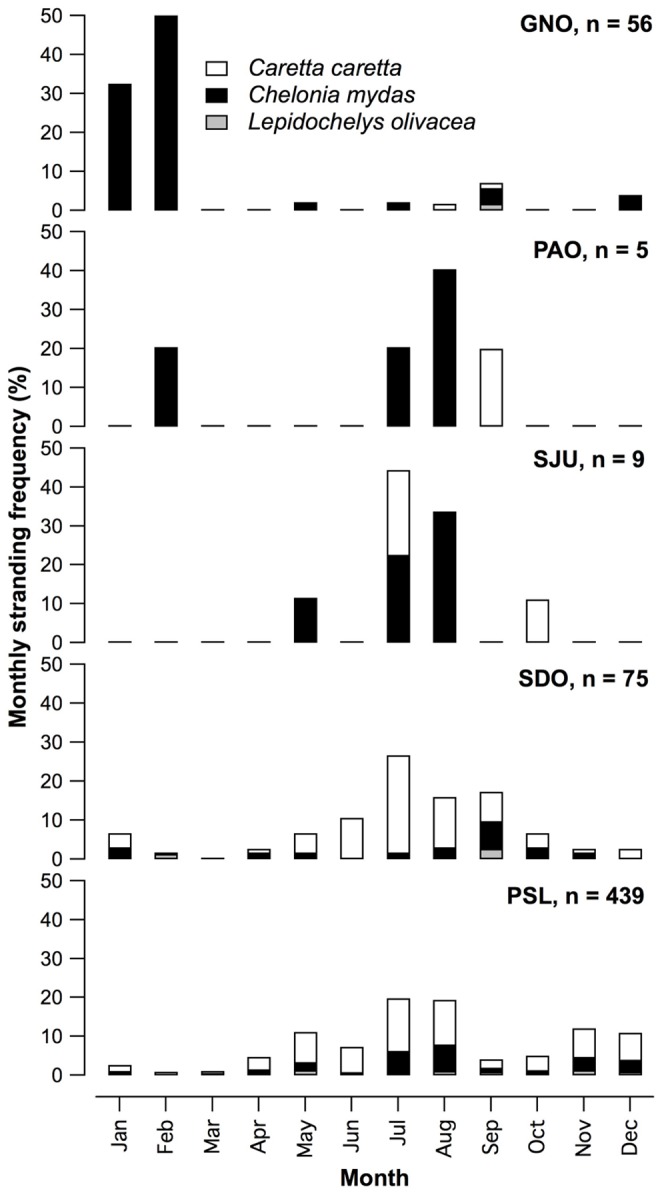
Monthly stranding frequencies of sea turtles in % of the total strandings at each site during 2010 and 2011. Site abbreviations are shown in Figure 1.

Monthly stranding frequencies showed a pronounced seasonal variation. Greater numbers of carcasses were found in July and August at all sites except GNO, where the number of strandings were greatest in January and February (Figure 2). At PSL, secondary peaks were found during November-December and May-June periods.

A total of 4752 individually marked drifters were deployed during nine trials, four at PSL (2010 and 2011), two each at SJU and PAO and one at GNO (all in 2010; Table 1).

**Table 1:** Deployed and retrieved drifters at each sampling location/trial, the mean stranding probability of drifters (*p*), 95% posterior probability intervals (PI) in square brackets, the number of observed stranded carcasses, and expected total deaths (15 days^−1^) of loggerhead (*Cc*), green (*Cm*), and olive ridley (*Lo*) turtles at four study sites along the Baja California peninsula.

Site	Latitude	Observation period	Drifter # Deployed/Retrieved	Cell sizes	*p* [95% PI]	# carcasses observed	Expected total deaths 15 d^−1^	[95% PI]
**PSL**	N: 25.1980°	9–23 July, 2010	1070/89	c	0.09	*Cc*: 6	44	[19, 87]
	S: 24.8370°			b	[0.04, 0.17]	*Cm*: 3	25	[7, 66]
	N: 25.1999°	29 July –12	1187/47	d	0.05	*Cc*: 13	88	[52, 139]
	S: 24.8946°	August, 2010		b	[0.02, 0.12]	*Cm*: 1	12	[1, 61]
	N: 25.1897°	25 July –8	261/93	b	0.36	*Cc*: 11	27	[17], [42]
	S: 24.9645°	August, 2011		b	[0.26, 0.47]	*Cm*: 11	27	[17], [42]
						*Lo*: 2	5	[2], [13]
	N: 25.1593°	3–17 October,	142/23	c	0.17	*Cc*: 2	10	[2], [32]
	S: 24.9964°	2011		b	[0.10, 0.26]			
**SJU**	N: 26.2617°	21 July –4	462/77	d	0.16	*Cc*: 2	14	[3, 60]
	S: 26.2150°	August, 2010		b	[0.02, 0.36]	*Cm*: 1	6	[1], [43]
	N: 26.2463°	6–21 August,	296/29	d	0.10	*Cm*: 2	15	[3], [49]
	S: 26.2309°	2010		b	[0.05, 0.19]			
**PAB**	N: 26.8069°	12–26 July,	374/52	c	0.13	*Cm*: 1	5	[1], [29]
	S: 26.7077°	2010		b	[0.07, 0.23]			
	N: 25.1980°	14–28 August,	454/250	b	0.53	*Cm*: 2	3	[2], [9]
	S: 24.8370°	2010		b	[0.22, 0.82]			
**GNE**	N: 28.0865°	22 July –5	506/394	b	0.79	*Cc*: 1	1	[1], [3]
	S: 27.9205°	August, 2010		b	[0.67, 0.87]	*Cm*: 1	1	[1], [3]

North and south latitudes correspond to the northern and southernmost latitudes at which drifters were recovered. Estimates of *p* and their 95% PI for 15-day periods are from the dataset in which drifter deployments were grouped into deployment (upper row) and retrieval (lower row) boxes (b  = 0.02°×0.02°, c  = 0.03°×0.03°, d  = 0.04°×0.04°). These estimates are from the binomial-Poisson model but other models provided similar results. The point estimate is the mode of the hyper-distribution of *p*’s for all boxes. See text for details.

Recovery rate ranged from less than 4% (PSL, August 2010) to over 75% (GNO August 2010), where the overall recovery rate was 22% (1054 oranges retrieved). Even within one location (PAO), recovery varied between trials from 14% in July to 55% in August. A difference in recovery rate between years also was found at PSL, where 4% were recovered in August 2010 but 36% were recovered in August 2011. For all locations, almost all stranded oranges were found within 10 days of deployment (Results not shown). One exception was for ALM in 2011, where oranges were located a few months after they were deployed offshore. However, more than 80% of all stranded oranges at ALM in 2011 were found stranded within 10 days of deployments. The cell size that provided the most precise estimate of the total deaths varied among locations (Table 1).

**Figure 3:**
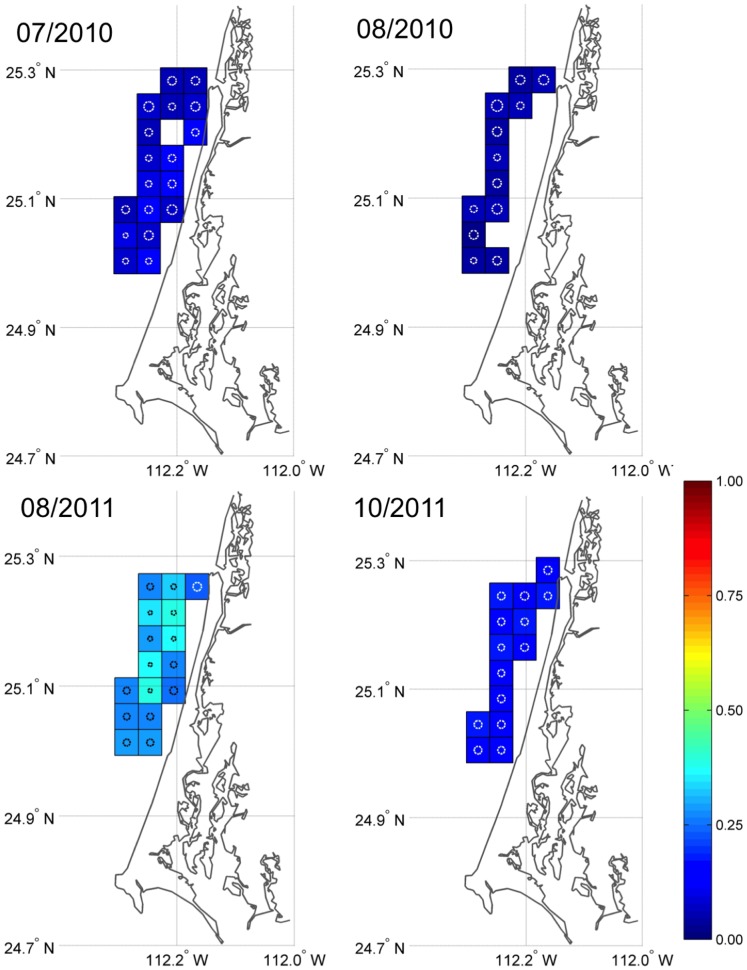
Estimated stranding probabilities for four drifter trials at the main fishing area in Playa San Lázaro (PSL) during 2010 and 2011. Estimates and their 95% posterior intervals are listed in Table 1. All deployment quadrats are 0.04×0.04 degrees. Circles in the quadrats are coefficient of variations (CV); a circle almost filling the quadrat corresponds to a CV of 1.0). The colored scale shows the stranding probability (p) indicating how likely oranges are to strand when deployed at different locations at sea.

**Figure 4:**
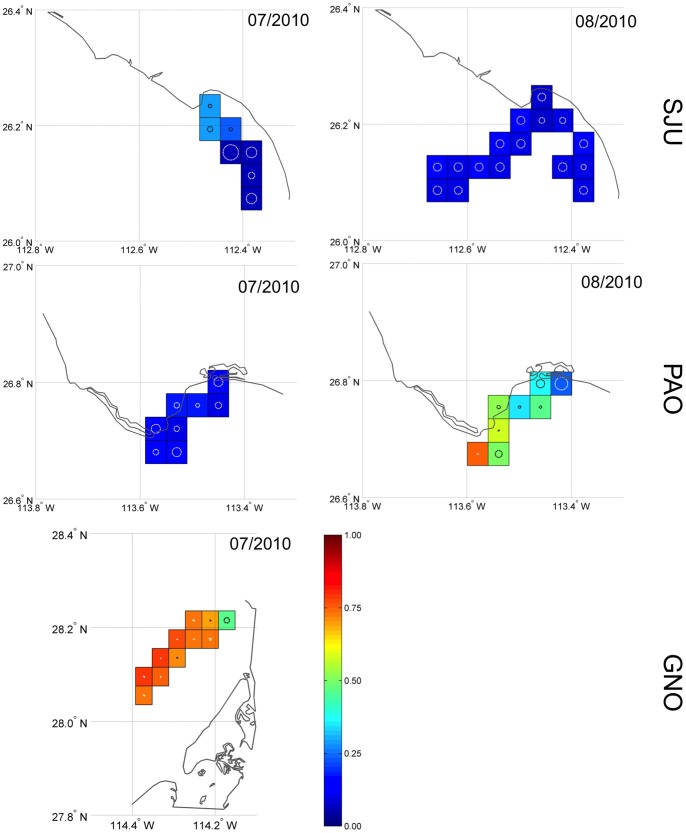
Estimated stranding probabilities for orange trials at the main fishing area in Guerrero Negro (GNO), **Punta Abreojos (PAO) and San Juanico (SJU) during 2010.** Estimates and their 95% posterior intervals are listed in Table 1. All deployment quadrats are 0.04×0.04 degrees. Circles in the quadrats are coefficient of variations (CV); a circle with half the diameter of the quadrat corresponds to a CV of 0.5). The colored scale shows the stranding probability (p) indicating how likely oranges are to strand when deployed at different locations at sea.

The probability of a drifter to strand when deployed at different points at sea (stranding probability, *p*) varied with no apparent patterns (Figures 3 and 4). Distance from shore did not seem to be a determining factor for *p* at the relatively small spatial scales (∼3–10 nm offshore) across which the experiment was conducted. Between study sites, stranding probabilities also varied considerably. At PSL, SJU, and PAO (only July 2010) stranding probability was less than 0.15 during 2010, whereas it was greater than 0.5 for PAO in August 2010 and even 0.8 for GNO. Stranding probability at PSL during August 2011 (0.36, 95%PI = [0.26, 0.47]) was greater than during August 2010 (0.05, 95%PI = [0.02, 0.12]; Table 1) and overall stranding probability was higher in 2011. Effects of the different prior distribution on the posteriors were negligible.

Logistical/financial constraints prohibited us from collecting local wind and current data simultaneously, and satellite-derived data for these near-shore environments are questionable. Due to the limited information on locations of fishing vessels (small skiffs operated by one or two fishers aboard), it was not possible to correlate spatial and temporal distributions of fishing effort and turtle/orange stranding probabilities in this study.

A comparison of posterior distributions for the two models for estimating the total number of carcasses (Binomial-Poisson and Negative Binomial) indicated that the estimated uncertainty from the Negative Binomial model was greater than for the Binomial-Poisson model. We, therefore, present results from the Binomial-Poisson model in the following. The posterior distributions of the hyper parameters of stranding probabilities (i.e., site-specific *p*) were unimodal, although the uncertainty around the parameter was large in some study areas (Table 1). The estimates of total mortality around the dates when the orange trials were conducted are presented in Table 1. By far the highest estimates 15 d^−1^ were calculated for PSL, with over 70 turtles (47 loggerhead 28 green) from the 9^th^ to 23^rd^ of July 2010 and over 100 turtles for August 2010 (90 loggerhead, 17 *C.* green). When the upper limits of the posterior intervals were considered, >150 turtles could have died during the 15 d period. This is remarkable as 2010 was the year with the lowest stranding numbers recorded since 2003 at PSL (2010 total: 204 strandings). During August and October 2011, estimated mortality was 64 (29 loggerhead, 29 green, 6 olive ridley) and 12 turtles (all loggerheads) in the same area (2011 total: 234 strandings). At the other sites a few to tens of sea turtles were estimated to have died during the experiments. Overall sea turtle mortality was lowest at GNO with an estimate of less than 5 turtles dying per 15 days.

## Discussion

Monthly stranding frequency of sea turtles was exceptionally high in PSL during 2010 and 2011 with 0.41 turtles km^−1^ month^−1^ followed by SDO, the barrier island just north of PSL, with 0.16 turtles km^−1^ month^−1^. These two sites alone accounted for 87% of all sea turtle strandings (and >95% of loggerhead strandings) while they represent only 1/3 of the total shoreline length sampled here. Sea turtle stranding rates at PSL are among the highest documented worldwide [4], [7], [12], [42]. Our results are consistent with previous studies by Peckham *et al*. [11], [12], and clearly underscore the importance of the area for conservation of loggerhead turtles.

Satellite telemetry [11] and aerial surveys (J. Seminoff, personal communication) have demonstrated that loggerhead turtles aggregate at the southern portion of the Gulf of Ulloa. Our observation recorded a large number of fishing vessels in the area. The large number of strandings in this region is therefore likely a result of the overlap of an aggregation of loggerheads and small-scale fisheries that frequently employ fishing gear with high bycatch rates, such as bottom-set nets and longlines [11], [12]. Interactions between turtles and medium and industrial scale fleets that operate in the hotspot have also been implicated [12]. Ocean currents, beach orientation, and the nearshore fishing area have been cited to favor stranding at PSL [3], [4], [15], but our results suggest otherwise as stranding probability *(p)* was mostly lower than at the other sites. The large difference between stranding probability at PAO may be explained by the occurrence of southerly swells in August 2010 (Mancini, pers. obs.).

The change in species dominance from south to north was also reported by Mancini and Koch [19], with loggerhead turtles being more abundant in the southern areas (see above) while green turtles are distributed more uniformly along the Pacific coast with a strong association to coastal lagoons, of which several are found further north (Figure 1) [43]. While the fishing seasons and target species change somewhat in the small-scale fisheries in BCS, most communities use the same fishing methods at similar depths [44]. The concentration of loggerhead strandings at PSL thus corresponds to the well-documented variation in loggerhead abundance along the coast rather than to spatial differences in fishing intensity, gear type and seasonality [11], [31]. Stranding data for leatherback turtles on the coasts of the Irish and Celtic Sea were similarly correlated with in-water distribution [45] and this approach has also been used to study the distribution of jellyfish [46].

Seasonal variation in stranding was pronounced at all sites, and summer months had the highest stranding frequency (except in GNO). This has also been described for the Mediterranean [8], [47], the Northwest Atlantic [3], the Hawaiian Archipelago [7] and the Eastern Pacific [12]. At our study sites, stranding frequency and fishing effort peak during the same months [12], [44]. Cause of death in most carcasses was impossible to determine due to advanced decomposition, scavenging, and the fact that sea turtles often show no easily recognizable marks from fishing gear [4], [8]. Indirect evidence, however, points towards artisanal coastal fisheries as the main source of mortality, as bycatch mortality is known to be a major problem in Bahia Magdalena [10], [25], in PSL [11], [12], in San Ignacio lagoon just south of PAO [4] and also at other sites in Baja California where interviews with fishermen revealed similar patterns [19], [25]. Additional sources of mortality from offshore industrial and medium-scale fishing activities are also likely to contribute to loggerhead mortality in the southern Gulf of Ulloa [12].

Strandings at GNO followed a different pattern with highest strandings in winter (January-February) when coastal gillnet and longline fisheries are closed in the area due to high grey whale (*Eschrichtius robustus*) abundance [44]. However, the site experiences cold spells during wintertime when easterly winds blow from the San Francisco mountain range into the coastal lowlands and air temperatures around freezing point can occur in Guerrero Negro (Exportadora de Sal, unpublished data). Green turtles that are hibernating in shallow waters may encounter lethal temperatures below 10°C [48], [49] during this time of the year. This phenomenon is currently under study, but the preliminary evidence and timing of cold spells and green turtle strandings suggests that both events are related.

We described in the methods section why we expected oranges and sea turtle carcasses to have similar drifting behavior at the surface. We had planned to compare drifting behavior of oranges and carcasses directly but due to the lack of accessible fresh carcasses, were unable to do it. One known difference between oranges and turtle carcasses is the initial sinking of freshly dead turtles and in consequence, their drifting behavior while they are negatively buoyant. For up to several days (depending on the water temperature), their density is greater than seawater, until decomposition produces enough gas to make positively buoyant. Using time-depth recorders on freshly deceased carcasses would allow to evaluate how they behave in the water column (see Hays et al. [50] for an application on jellyfish).

Our stranding probability (*p*) estimates varied widely, and while most were in the range of 0.05–0.20, as expected from earlier studies [2], [3], [4], others were notably higher. For example, it was 0.80 at GNO, which is an excellent area for beachcombing. We have found during our beach monitoring much debris originating from the Western Pacific, such as glass buoys, litter with Chinese and Japanese writings, and other artifacts. PAO also had a high p of 0.52 during August of 2010, when southerly winds and swells dominated (Mancini, pers. obs.). The large difference found between consecutive trials in PSL during 2011, and especially in PAO in 2010 show that the conditions that determine stranding probability may change quickly on small spatial scales. Both, the large differences in p between study sites and the large temporal differences in p at the same site (up to fourfold) have important implications for the interpretation of stranding frequencies. Stranding data are the most easily accessible data for inferring at-sea mortality. They also can provide information on causes of deaths. Further, these may be the only information available for rare species [1]. However, they do not provide reliable estimates on mortality at sea without ancillary data [51] and they are not directly comparable between different sites, nor over time at the same site, due to large spatiotemporal variations in stranding probability. Casale et al. [8] proposed to study strandings over long stretches of coastline (>100 km) to account for small-scale differences in stranding probability. This approach (where feasible) is a great improvement, but it still fails to account for large differences in stranding probabilities over time that may severely bias interpretation of stranding frequencies and the probable causes of mortality and impacts from fisheries or other events. However, in the absence of other metrics, the relative abundance of sea turtles strandings over time may still provide a very useful index for determining trends.

In our trials, distance from shore did not seem to be closely correlated to stranding probability, contrary to results reported by Hart *et al*. [3]. Our drifter trials were conducted to provide effective coverage of the respective fishing areas only, and therefore we did not go further than 5–10 nm from the coast. Preliminary experiments on a larger scale (up to 20 nm offshore, Peckham, unpubl. data), have demonstrated this relationship also for PSL, as can be expected. This also means that the artisanal shark fishery in BCS that is operating at 20–40 nm offshore with longlines and gillnets may have large impacts on turtle and marine mammal populations, although few if any of the carcasses generated by this fishery are likely to strand. Observations from local fishermen indicate high bycatch rates in this fishery (Peckham, pers. obs.).

Our mortality estimates during the 15-day periods around our trials show that the southern Gulf of Ulloa (PSL) continues to be an important hot spot for loggerhead turtle mortality [11], [12]. Mortality estimates only from the two trials in 2010 (30 days) almost reached the total of strandings during the same year, probably causing a massive impact on the North Pacific loggerhead population [12]. Fortunately, mortality estimates at the other sites are much lower, but our study did not cover the area between Lopez Mateos and south of SJU, where several other fishing fleets operate. The stranding results from SDO just north of PSL together with the results from Peckham et al. [12] on the massive impact of a small longline fleet (6 boats) operating north of SDO indicate that the problem is probably occurring in other parts of the Gulf of Ulloa as well. Our first quantitative estimates of mortality at sea for different sea turtle species from four fishing areas provide a reasonable approximation, but we suggest several possible improvements for future studies. We did not include spatial correlations of stranding probabilities in the model and stranding probabilities could be modeled as source-destination pairs. It is possible that carcasses in some areas are more likely to end up in particular locations, rather than randomly reaching the shore. Although we tried to tease out such possibility by using unique coding on all drifter buoys, some codes became illegible during the experiment thereby reducing the number of usable drifters for such modeling.

When current data are available for a particular region [39], particle tracking models (PTMs) are very useful to predict the currents influencing sea turtles (or their carcasses). This approach has been used to elucidate the behavior of satellite tracked leatherback turtles in relation to prevailing currents [52] and also to backtrack the origin of loggerhead turtles stranded in France [53]. The latter approach would have been our method of choice, but no current data were available to cover near-shore waters on a sufficiently small spatial and temporal scale. For future applications, we recommend combining drifter data (source-destination pairs) with oceanographic data, and conducting drifter experiments in parallel with artificial turtles (similar shape, size and density) to see if the assumption holds that their surface drift behavior is similar to oranges. Ideally, individual drift paths of turtles and drifters would be followed using low-cost waterproof GPSs, which would greatly increase the precision of stranding probability and death at sea estimates. Use of VHF transmitters may increase the recovery of drifters and carcasses and turtle carcasses should always be deployed with TDRs to record how long and how deep they stay underwater after death.

While observer programs for small-scale fisheries can offer comprehensive data on fishery-related turtle bycatch data, they can be prohibitively expensive in both financial and sociopolitical terms [12]. This is mostly due to the difficulties inherent to systematically working with a large, dispersed, and little-regulated fleet, especially in developing countries [54]. Further, small skiffs (6–8 m length) used in these fishing operations may not have enough room for observers. Therefore, our approach may be more feasible in many cases.

## References

[1] Geraci JR, Lounsbury VJ (2005) Marine Mammals ashore: A field guide for strandings, second edition. Baltimore: National Aquarium in Baltimore. 380 p.

[2] Epperly SP, Braun J, Chester AJ, Cross FA, Merriner JV, et al (1996) Beach strandings as an indicator of at-sea mortality of sea turtles. Bull Mar Sci 59: 289–297.

[3] Hart KM, Mooreside P, Crowder LB (2006) Interpreting the spatio-temporal patterns of sea turtle strandings: going with the flow. Biol Conserv 129: 283–290.

[4] Mancini A, Koch V, Seminoff JA, Madon B (2011) Small-scale gill-net fisheries cause massive green turtle *Chelonia mydas* mortality in Baja California Sur, Mexico. Oryx 46(1): 69–77.

[5] Geraci JR, Harwood J, Lounsbury VJ (1999) Marine Mammal die-offs. Causes, investigations and issues. In: Conservation and management of marine mammals. Twiss JR, Reeves RR, editors. Washingon DC: Smithsonian Institution Press. 367–396.

[6] Lewison RL, Crowder LB, Read AJ, Freeman SA (2004) Understanding impacts of fisheries bycatch on marine megafauna. Trends Ecol Evol 19: 598–604.

[7] Chaloupka M, Work TM, Balazs GH, Murakawa SKK, Morris R (2008) Cause-specific temporal and spatial trends in green sea turtle strandings in the Hawaiian Archipelago (1982–2003). Mar Biol 154: 887–898.

[8] Casale P, Affronte M, Isacco G, Freggi D, Vallini C, et al (2010) Sea turtle strandings reveal high anthropogenic mortality in Italian waters. Aquat Conserv 20: 611–620.

[9] IUCN (2012) IUCN Red List of Threatened Species. Available: http://www.iucnredlist.org/. Accessed 12 July 2012.

[10] Koch V, Nichols WJ, Peckham H, De La Toba V (2006) Estimates of sea turtle mortality from poaching and bycatch in Bahía Magdalena, Baja California Sur, Mexico. Biol Conserv 128: 327–334.

[11] Peckham SH, Diaz DM, Walli A, Ruiz G, Crowder LB, et al (2007) Small-scale fisheries bycatch jeopardizes endangered pacific loggerhead turtles. PLoS ONE 2(10): e1041 doi:10.1371/journal.pone.0001041. 17940605 10.1371/journal.pone.0001041 PMC2002513

[12] Peckham SH, Maldonado-Diaz D, Koch V, Mancini A, Gaos A, et al (2008) High mortality of loggerhead turtles due to bycatch, human consumption and strandings at Baja California Sur, Mexico, 2003 to 2007. Endanger Species Res 5: 171–183.

[13] Read A (2008) The looming crisis: interactions between marine mammals and fisheries. J Mammal 89 (3): 541–548.

[14] Wallace BP, Lewison RL, McDonald S, McDonald R, Cot CY, et al (2010) Global patterns of marine turtle bycatch. Conservation letters 3(2): 1–12.

[15] Mercuri M (2008) Varamientos de mamiferos marinos en Isla Magdalena, BCS Mexico y su relación con factores físicos y bíologicos. Master Thesis, La Paz: Instituto Politecnico Nacional-CICIMAR. 125 p.

[16] Mancini A, Senko J, Borquez-Reyes R, Pόo JG, Seminoff JA, et al (2011) To poach or not to poach an endangered species: Elucidating the economic and social drivers behind illegal sea turtle hunting in Baja California Sur, Mexico. Hum Ecol 39: 743–756.

[17] Nichols WJ, Safina C (2004) Lunch with a Turtle Poacher. Conservation in Practice 5(4): 30–36.

[18] Koch V, Nichols WJ, Brooks LB (2007) Population ecology of the green/black turtle (*Chelonia mydas*) in Bahía Magdalena, Mexico. Mar Biol 153(1): 35–46.

[19] Mancini A, Koch V (2009) Sea turtle consumption and black market trade in Baja California Sur, Mexico. Endanger Species Res 7: 1–10.

[20] Godley BJ, Gücü AC, Broderick AC (1998) Interaction between marine turtles and artisanal fisheries in the eastern Mediterranean: a probable cause for concern? Zool Middle East 16: 49–84.

[21] Alfaro-Shigueto J, Mangel J, Seminoff JA, Dutton PH (2008) Demography of loggerhead turtles *Caretta caretta* in the southeastern Pacific Ocean: fisheries-based observations and implications for management. Endanger Species Res 5: 129–135.

[22] Alfaro-Shigueto J, Mangel JC, Bernedo F, Dutton PH, Seminoff JA, et al (2011) Small-scale fisheries of Peru: a major sink for marine turtles in the Pacific. J Appl Ecol 48(6): 1432–1440.

[23] Hamann M, Godfrey MH, Seminoff JA, Arthur K, Barata PCR, et al (2010) Global research priorities for sea turtles: informing management and conservation for the 21^st^ century. Endanger Species Res 11: 245–269.

[24] Soykan CU, Moore JE, Zydelis R, Crowder LB, Safina C, et al (2008) Why study bycatch? An introduction to the Theme Section on fisheries bycatch. Endanger Species Res 5: 91–102.

[25] Nichols WJ (2003) Biology and Conservation of the Sea Turtles of the Baja California Peninsula, Mexico. PhD Dissertation. Tucson: University of Arizona. 488 p.

[26] Moore JE, Cox TM, Lewison RL, Read AJ, Bjorkland R, et al (2010) An interview- based approach to assess marine mammal and sea turtle captures in artisanal fisheries. Biol Conserv 143: 795–805.

[27] Petersen SL, Phillips RA, Ryan PG, Underhill LG (2008) Albatross overlap with fisheries in the Benguela Upwelling System: implications for conservation and management. Endanger Species Res 5: 117–127.

[28] Prellezo R, Gallastegui MC (2008) Gear-selectivity-based regulation in a mixed fishery. Endanger Species Res 5: 335–344.

[29] Gilman E (Editor) (2009) Proceedings of the Technical Workshop on Mitigating Sea Turtle Bycatch in Coastal Net Fisheries. 20–22 January 2009, Honolulu, U.S.A. Western Pacific Regional Fishery Management Council, IUCN, Southeast Asian Fisheries Development Center, Indian Ocean – South-East Asian Marine Turtle MoU, U.S. National Marine Fisheries Service, Southeast Fisheries Science Center: Honolulu; Gland, Switzerland; Bangkok; and Pascagoula, USA.

[30] Mangel JC, Alfaro-Shigueto J, Van Waerebeek K, Caceres C, Bearhop S, et al (2010) Small cetacean captures in Peruvian artisanal fisheries: high despite protective legislation. Biol Conserv 143: 136–143.

[31] Peckham SH, Maldonado-Diaz D, Tremblay Y, Ochoa R, Polovina J, et al.. (2011) Demographic implications of alternative foraging strategies in juvenile loggerhead turtles Caretta caretta of the North Pacific Ocean. Mar Ecol Prog Ser 425: 269–280. doi 10.3354/meps08995.

[32] Wingfield D, Peckham SH, Foley DG, Palacios DM, Lavaniegos BE, et al.. (2011) The making of a predator hotspot in the coastal ocean. PLOS One 6(11): e27874 doi 10.1371/journal.pone.0027874. 10.1371/journal.pone.0027874 PMC3221696 22132156

[33] Zaytsev O, Cervantes-Duarte R, Montante O, Gallegos-Garcia A (2003) Coastal upwelling activity on the Pacific shelf of the Baja California peninsula. J Oceanogr 59: 489–502.

[34] Espinosa-Carreon TL, Strub PT, Beier E, Ocampo-Torres F, Gaxiola-Castro G (2004) Seasonal and interannual variability of satellite-derived chlorophyll pigment, surface height, and temperature off Baja California. J Geophys Res 109: doi:03010.01029/02003JC002105.

[35] Legaard KR, Thomas AC (2006) Spatial patterns in seasonal and interannual variability of chlorophyll and sea surface temperature in the California Current. J Geophys Res 111: doi:10.1029/2005JC003282.

[36] Secretaría de Agricultura, Ganadería, Desarrollo Rural, Pesca y Alimentación (SAGARPA) (2010) Anuario de pesca. Available: http://www. sagarpa.gob.mx. Accessed: 19 July 2012.

[37] INEGI (2005) Anuario estadistico del estado de Baja California Sur. Available: http://www.inegi.gob.mx. Accessed: 19 July 2012.

[38] Sharifi M, Rafiee S Keyhani A, Jafari A, Mobli H, et al (2007) Some physical properties of orange (var. Tompson). Int Agrophysics 21: 391–397.

[39] Fossette S, Putman NF, Lohmann KJ, Marsh R, Hays GC (2012) A biologist’s guide to assessing ocean currents: a review. Mar Ecol Prog Ser Vol. 457: 285–301.

[40] Raftery AE (1988) Inference for the binomial N parameter: A hierarchical Bayes approach. Biometrika 75: 223–228.

[41] Lunn D, Spiegelhalter D, Thomas A, Best N (2009) The BUGS project: Evolution, critique, and future directions. Stat Med 28: 3049–3067. 19630097 10.1002/sim.3680

[42] Shanker K, Pandav B, Choudhury BC (2004) An assessment of the olive ridley turtle (Lepidochelys olivacea) nesting population in Orissa, India. Biol Conserv 115: 149–160.

[43] Lopez-Castro MC, Koch V, Mariscal-Loza A, Nichols WJ (2010) Long-term monitoring of black turtles *Chelonia mydas* at coastal foraging areas off the Baja California Peninsula. Endanger Species Res 11: 35–45.

[44] Mancini A (2009) Pesca incidental o captura dirigida – tasas y causas de mortalidad de tortugas marinas en BCS, Mexico. PhD Dissertation. La Paz: Universidad Autónoma de BCS, Mexico.

[45] Houghton JDR, Doyle TK, Wilson MW, Davenport J, Hays GC (2006) Jellyfish aggregations and leatherback turtle foraging patterns in a temperate coastal environment. Ecology 87: 1967–1972. 16937635 10.1890/0012-9658(2006)87[1967:jaaltf]2.0.co;2

[46] Doyle TK, Houghton JDR, Buckley SM, Hays GC, Davenport J (2007) The broad-scale distribution of five jellyfish species across a temperate coastal environment. Hydrobiologia 579 29–39. doi: 10.1007/s10750-006-0362-2.

[47] Tomás J, Gozalbes P, Raga JA, Godley BJ (2008) Bycatch of loggerhead sea turtles: insights from 14 years of stranding data. Endang Species Res 5: 161–169.

[48] Witherington B, Ehrhart LM (1989) Hypothermic stunning and mortality of marine turtles in the Indian River Lagoon system, Florida. Copeia 1989(3): 696–703.

[49] Shaver JD (1990) Hypothermic Stunning of Sea Turtles in Texas. MTN 48: 25–27.

[50] Hays GC, Bastian T, Doyle TK, Fossette S, Gleiss AC, et al (2012) High activity and Lévy searches: jellyfish can search the water column like fish. Proc Royal Soc London B. 279: 465–473 doi:10.1098/rspb.2011.0978. 10.1098/rspb.2011.0978 PMC3234559 21752825

[51] Eguchi T (2002) A method for calculating the effect of a die-off from stranding data. Mar Mam Sci 18(3): 698–709.

[52] Hays GC, Fossette S, Katselidis KA, Mariani P, Schofield G (2010) Ontogenetic development of migration: Lagrangian drift trajectories suggest a new paradigm for sea turtles. J Royal Soc Interface 7: 1319–1327 doi:10.1098/rsif.2010.0009. 10.1098/rsif.2010.0009 PMC2894886 20236958

[53] Monzon-Arguello C, Dell’Amico F, Morinière P, Marco A, López-Jurado LF, et al.. (2012) Lost at sea: genetic, oceanographic and meteorological evidence for storm-forced dispersal. J Royal Soc Interface, doi:10.1098/rsif.2011.0788. 10.1098/rsif.2011.0788 PMC3385747 22319111

[54] Lewison R, Soykan C, Cox T, Peckham H, Pilcher N, et al (2011) Ingredients for addressing the challenges of fisheries bycatch. Bulletin of Marine Science 87 (2): 235–250.

